# Vibration as a pitfall in pyrosequencing analyses

**DOI:** 10.1007/s00414-021-02716-7

**Published:** 2021-10-12

**Authors:** Helen Konrad, Laura Schäfer, Hannah Sturm, Lena Hördt, Thomas Bajanowski, Micaela Poetsch

**Affiliations:** Institute of Legal Medicine, University Hospital Essen, Hufelandstr 55, 45122 Essen, Germany

**Keywords:** Methylation, Methylation analysis, Pyrosequencing, Vibration, CpGs

## Abstract

Since methylation analysis has become an important tool in forensic genetics, the reliability and credibility of the method must be ensured. After a successful validation and establishment of several pyrosequencing assays using a PyroMark® Q48 Autoprep instrument (Qiagen, Hilden, Germany), we decided to expand the method further purchasing a second instrument. But after initializing this second instrument side by side with the first, the majority of analyses failed (97 samples of 133 samples (73%)). The number of error messages increased rapidly and the average RFU values decreased. After purchasing two anti-vibration weighing tables for the PyroMark® instruments and repeating the analyses under the same conditions and with identical samples the results improved considerably, 115 samples of 130 samples (88%) showed successful and reproducible results. These findings demonstrate the impact of vibrations and percussions on PyroMark® Q48 Autoprep performance and the reliability of methylation analyses.

**Supplementary Information:**

The online version contains supplementary material available at 10.1007/s00414-021-02716-7.

## Introduction

DNA methylation analysis is a method with increasing importance in forensic genetic research and casework [1, 2]. Especially, estimation of biological age by analysis of age-dependent CpG sites, but also body fluid identification by determining the methylation status of certain CpGs, has become a relevant tool [reviewed by 3, 4]. Several techniques could be employed for methylation analysis, e.g., *massive parallel sequencing* (MPS) [5], *methylation-specific-PCR* (MSP) [6], or pyrosequencing [7]. The reliability and credibility of results produced by these methods depend on the quality of the established assays as well as on the performance of the employed instruments. Here, we describe the impact of vibrations on the meaningfulness of results produced by a PyroMark® Q48 Autoprep (Qiagen, Hilden, Germany). For this purpose, identical samples have been analyzed under three different conditions.

## Material and methods

### Samples

The study comprised saliva, blood, and menstrual blood samples from different individuals of different age. All in all, 130 samples were collected in 2020 and 2021 in the Institute of Legal Medicine, University Hospital Essen, Germany.

### DNA extraction, quantification, bisulfite conversion, amplification, and sequencing

DNA extraction was performed using DNA IQ Casework Pro Kit and Casework Extraction Kit in the Maxwell 16® instrument according to the manufacturer’s instructions (Promega, Mannheim, Germany), resulting in an extraction volume of 50 µl. DNA concentration of samples was established by real-time PCR using the PowerQuant™ System (Promega) according to the manufacturer’s instructions providing a reproducible and reliable detection threshold at least down to 25 pg DNA [8]. Using 2 µl DNA-containing solutions, each sample was analyzed in duplicates. Bisulfite conversion was performed applying MethylEdge Conversion System Kit (Promega) corresponding to the manufacturer’s instructions with an increased elution volume of 20 µl. An initial DNA amount of 50 ng was used in the conversion. DNA amplification of age estimation CpGs [9] as well as candidate CpGs for body fluid identification [10, 11] was done using PyroMark® PCR Kit (Qiagen) following the manufacturer’s instructions, adapted to an increased number of 50 cycles. One of the two PCR primers was biotinylated.

Sequence analysis was established in a PyroMark® Q48 Autoprep instrument using the PyroMark® Q48 Advanced CpG Reagent Kit according to the manufacturer’s instructions (Qiagen). Every sample and CpG site was analyzed at least twice.

### Experimental setups

In the first setup, only one PyroMark® Q48 Autoprep instrument placed on a normal working bench was employed. In the second setup, another PyroMark® Q48 Autoprep instrument was installed side by side with the first one and both instruments ran simultaneously on a normal working bench. In the final setup, both instruments ran simultaneously in close proximity, but each one was installed on a separate anti-vibration weighing table. All 130 samples have been analyzed in every setup.

## Results and discussion

### Reliability of data and DNA concentrations

Due to the demand of downstream methods, especially bisulfite conversion, all samples included in this study showed a DNA concentration between 2.5 and 50 ng/µl. For all three analysis setups, the same 130 samples were used, so that an impact of incomplete bisulfite conversion or differences in sample quality can be excluded.

### Evaluation strategy

The instrument has three different quality parameters: *failed*, *check*, and *passed*. If a sample displayed a result in the category *failed*, it cannot be evaluated in most cases. Several error messages, a very low RFU (mean value 48 ± 39; see Table S1 for details), and a baseline drift typically occur in these samples. A sample with the result *check* is usually reliable. Nevertheless, there may still be error messages and only a medium RFU height (mean value 77 ± 65; see Table S1 for details), but generally, no baseline drifts are present. The best result is category *passed* that normally indicates high RFU values (mean value 114 ± 47; see Table S1 for details) without error messages.

The software generates two different categories of error messages to convey problems during analysis, *general warnings* and *positions warnings*.

*General warnings* usually appear in systematic problems, e.g., because of failed bisulfite conversion or a dispensation of drops with unusual shape.

*Positions warnings* indicate position-related problems, e.g., too low or too high peak height. Moreover, a baseline drift also may result in a *positions warning*.

### Sequence analysis

In the first setup, one instrument PyroMark® Q48 Autoprep (Qiagen) was used and several assays in context of age estimation could be established and validated [9]. More than three quarter of analyses demonstrated reliable and reproducible results categorized as either *check* or *passed* by the instrument software (Table 1). Nevertheless, we observed a few error messages and a rather low median RFU height (78 ± 65), but generally, no baseline drifts and warnings were present (Table S1).

**Table 1:** Evaluation of pyrograms after sequence analysis in three different setups

Sequence analysis quality	One instrument	Two instruments side by side	Anti-vibration weighing table
	(*n* = 130)	(*n* = 130)	(*n* = 130)
*Passed*	42 (32%)	17 (13%)	**115 (88%)**
*Check*	**59 (45%)**	16 (12%)	12 (9%)
*Failed*	29 (22%)	**97 (75%)**	3 (2%)

Deviations from 100% are caused by rounding. For each setup, values in the category with the majority of results are printed in bold

In order to expand this kind of analysis and to establish further assays in context of body fluid analysis, a second PyroMark® Q48 Autoprep (Qiagen) was purchased. This was installed by a Qiagen’s technician side by side with the first instrument. He confirmed that this placement would not lead to any problems with data evaluation. However, using both instruments simultaneously side by side (setup 2), the main part of samples in both devices was qualified as *failed* (97 samples of 130 samples (75%) (Table 1)). The number of error reports and baseline drifts increased rapidly and the average RFU decreased considerably (Table S1). Looking for possible causes, we found a very short hint at possible problems with vibrations on page 29, 4.2.1 *Installation site* of the PyroMark® Q48 Autoprep User Manual [12]. Since vibrations are also a known problem for other high-end instruments (e.g., MiSeq FGx™ Instrument, 13), two anti-vibration weighing tables (Bosche Wägetechnik, Damme, Germany) were purchased (Figure S1). A heavy stone, balanced on rubber, protects the PyroMark® Q48 instrument from vibrations transmitted through the floor or along the working bench.

After performing sequence analysis with both instruments placed on anti-vibration weighing tables (setup 3), the results improved considerably and surpassed even those of our first experiments. More than 97% of analyses were evaluated as *check* or *passed* (Table 1). In the majority of results, pyrograms showed no warnings or baseline drifts and high RFUs could be reached especially in the category *passed* (Table S1).

The manual of the PyroMark® Q48 suggests a lot of possible sources for a bad quality of results/pyrograms, e.g., failed bisulfite conversion, a dispensation of drops with unusual shape, or too low/high peak height. However, the results of this study propose vibration as the main error source, since the presence or absence of vibration is the only difference between the three experimental setups due to use of identical samples, reagents, and procedures. Moreover, for setup one, no differences between the two instruments could be observed (data not shown). The principle of pyrosequencing is based on a locus and time-dependent detection of light (chemo luminescence). Therefore, it is understandable that any moving of the instrument during a run has an impact on the quality of the results. Due to occurrence of vibration, time and locus could be (slightly) out of alignment and the time- and locus-dependent detection fails. As a result, the expected RFU values will not be achieved. Due to the increasing number of *general warnings*, it can be concluded that the injectors are not performing properly under the influence of vibrations and may produce drops of unusual shape.

## Conclusion

The results of this study clearly demonstrate that vibration is a major problem for pyrosequencing instruments leading to unreliable and not reproducible outcomes. We recommend a more assertive description of these phenomena in the manual of the instrument, probably including the advice to install the devices only in vibration-free environments.

All samples were obtained after informed consent and with approval of the Medical Ethics Committee at the University of Duisburg-Essen in accordance with the Declaration of Helsinki and national laws (ethic vote numbers: 16–7113-BO, 21–9843-BO).

## Supplementary Information

Below is the link to the electronic supplementary material.

Supplementary file1 (DOCX 2713 KB)

Supplementary file2 (DOCX 14 KB)
