# Supplementary figures and images for: Vibration as a pitfall in pyrosequencing analyses
Source: Int J Legal Med. 2021 Oct 12;136(1):103–5. doi: 10.1007/s00414-021-02716-7 (PMC8813862; doi:10.1007/s00414-021-02716-7)

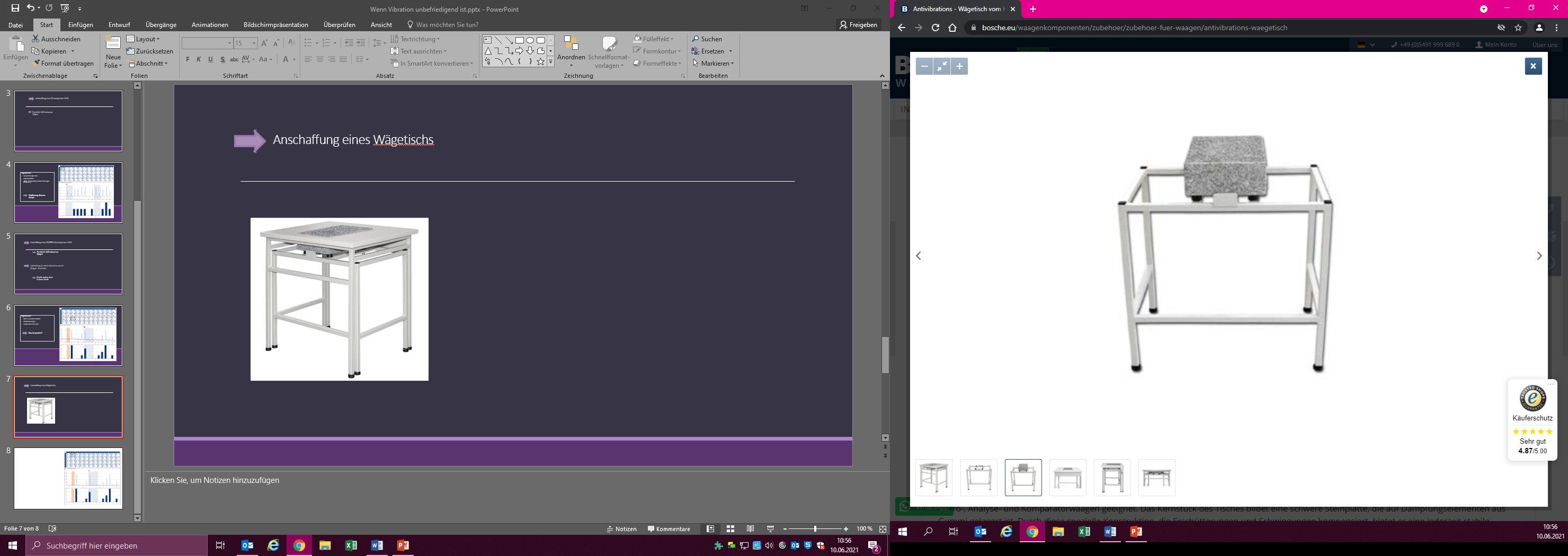

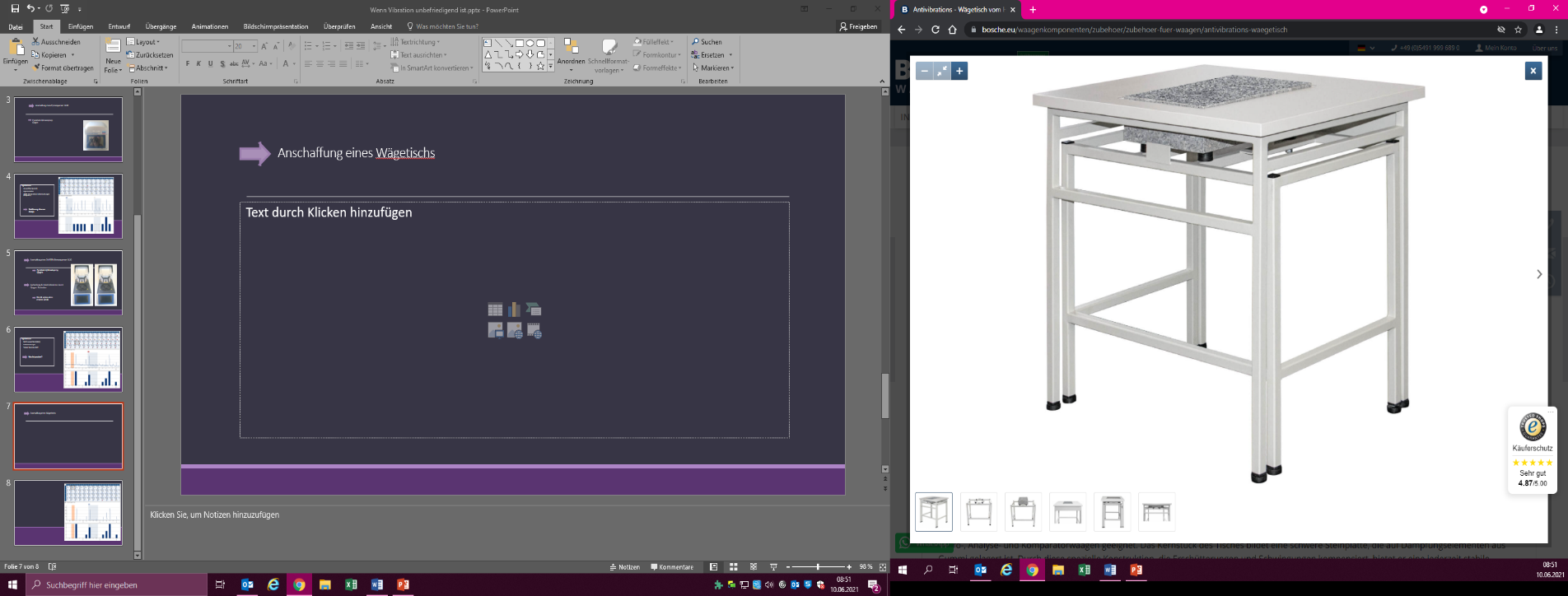


**Figure S1: Anti-vibration weighing table (Bosche Wägetechnik, Damme, Germany)**

Supplement: Supplementary file 1 — Supplementary file1 (DOCX 2713 KB) [file 414_2021_2716_MOESM1_ESM.docx]
