# Supplementary material for: Vibration as a pitfall in pyrosequencing analyses
Source: Int J Legal Med. 2021 Oct 12;136(1):103–5. doi: 10.1007/s00414-021-02716-7 (PMC8813862; doi:10.1007/s00414-021-02716-7)
Supplement: Supplementary file 2 — Supplementary file2 (DOCX 14 KB) [file 414_2021_2716_MOESM2_ESM.docx]

**Table S1: Additional quality parameters of the 130 analyzed samples sorted according to quality categories of the software and set ups**

|  | **one instrument**  **(set up 1)** | **two instruments side by side**  **(set up 2)** | **anti-vibration weighing table**  **(set up 3)** |
| --- | --- | --- | --- |
| **RFU median values** |  |  |  |
| All samples | 75 | 40 | 100 |
| Samples categorized as *passed* | 75 | 100 | 125 |
| Samples categorized as *check* | 75 | 75 | 45 |
| Samples categorized as *failed* | 35 | 25 | 30 |
| **RFU Ø ± standard deviation** |  |  |  |
| All samples | 78 ± 65 | 49 ±39 | 114 ± 47 |
| Samples categorized as *passed* | 83 ± 44 | 91 ± 19 | 123 ±41 |
| Samples categorized as *check* | 92 ± 81 | 79 ± 28 | 48 ± 16 |
| Samples categorized as *failed* | 43 ± 35 | 35 ± 34 | 33 ± 21 |
| **Samples with baseline drift** |  |  |  |
| Samples categorized as *passed* | 0 | 0 | 0 |
| Samples categorized as *check* | 1 | 5 | 10 |
| Samples categorized as *failed* | 11 | 64 | 2 |
| **Samples with general warnings** |  |  |  |
| Samples categorized as *passed* | 16 | 35 | 2 |
| Samples categorized as *check* | 133 | 37 | 0 |
| Samples categorized as *failed* | 48 | 261 | 0 |
| **Samples with position warnings** |  |  |  |
| Samples categorized as *passed* | 2 | 0 | 0 |
| Samples categorized as *check* | 58 | 26 | 18 |
| Samples categorized as *failed* | 48 | 244 | 7 |
